# Supplementary material for: Variations in the Mood States during the Different Phases of COVID-19’s Lockdown in Young Athletes
Source: Int J Environ Res Public Health. 2021 Sep 3;18(17):9326. doi: 10.3390/ijerph18179326 (PMC8430794; doi:10.3390/ijerph18179326)
Supplement: Supplementary file 1 [file ijerph-18-09326-s001.zip › ijerph-1299011-supplementary.pdf]

Table S1. Summary of the mood state scores of both sex at the different time points.

Outcome		Week 1	Week 6	Week 10
TMD (AU)				
	Male	124.71 ± 34.37	120.07 ± 27.91	114.69 ± 28.85
	Female	129.17 ± 24.37	124.23 ± 29.41	119.07 ± 29.89
Tension (AU)				
	Male	9.29 ± 5.38	8.50 ± 5.61	8.77 ± 5.48
	Female	10.57 ± 5.96	9.03 ± 5.62	7.74 ± 5.97
Depression (AU)				
	Male	9.57 ± 11.32	6.79 ± 7.49	4.85 ± 8.08
	Female	8.90 ± 7.07	7.37 ± 7.88	6.30 ± 7.66
Anger (AU)				
	Male	9.71 ± 8.16	7.93 ± 6.94	6.69 ± 7.16
	Female	9.87 ± 7.51	9.83 ± 8.45	8.78 ± 8.09
Vigor (AU)				
	Male	15.57 ± 6.28	14.50 ± 6.06	15.23 ± 5.72
	Female	14.37 ± 5.01	14.23 ± 4.70	14.89 ± 3.85
Fatigue (AU)				
	Male	4.57 ± 4.05	4.36 ± 3.67	3.69 ± 3.47
	Female	5.83 ± 4.32	5.57 ± 4.35	5.04 ± 4.97
Confusion (AU)				
	Male	7.14 ± 4.90	7.00 ± 5.13	5.92 ± 5.41
	Female	8.37 ± 4.57	7.47 ± 4.44	6.11 ± 4.90

Data are presented as mean ± SD. TMD: Total Mood Disturbance. AU: Arbitrary units.

Table S2. Summary of the mood state scores of the 3 sports at the different time points.

Outcome		Week 1	Week 6	Week 10
TMD (AU)				
	Athletics	119.28 ± 24.18	112.39 ± 28.34	106.22 ± 23.09
	Handball	136.94 ± 26.62#	139.81 ± 32.33#	135.54 ± 33.26#
	Swimming	128.30 ± 32.44	115.64 ± 19.38	114.67 ± 23.60
Tension (AU)				
	Athletics	10.11 ± 6.44	8.06 ± 4.52	6.89 ± 4.97
	Handball	11.38 ± 5.51	11.80 ± 6.49	10.08 ± 7.03
	Swimming	8.30 ± 4.76	6.18 ± 4.12^	7.56 ± 5.05
Depression (AU)				
	Athletics	7.17 ± 7.14	4.50 ± 6.78	2.83 ± 6.46
	Handball	10.56 ± 8.16	11.60 ± 8.76	10.85 ± 8.51#
	Swimming	10.30 ±11.26	5.55 ± 4.74	4.56 ± 5.55
Anger (AU)				
	Athletics	8.00 ± 6.32	6.50 ± 5.71	5.22 ± 5.33
	Handball	12.00 ± 8.15	14.53 ± 9.32#	12.69 ± 9.99
	Swimming	9.60 ± 8.73	6.45 ± 5.66^	7.22 ± 5.52
Vigor (AU)				
	Athletics	16.11 ± 5.44	15.94 ± 5.21	16.17 ± 4.97
	Handball	13.81 ± 5.15	12.87 ± 5.08	13.39 ± 3.53
	Swimming	13.80 ± 5.75	13.64 ± 4.59	15.00 ± 4.33
Fatigue (AU)				
	Athletics	3.83 ± 3.24	3.94 ± 3.13	3.00 ± 2.61
	Handball	6.56 ± 4.63	7.20 ± 4.75	6.31 ± 5.60
	Swimming	6.50 ± 4.60	4.46 ± 4.01	5.33 ± 5.27
Confusion (AU)				
	Athletics	6.28 ± 3.75	5.33 ± 4.45	4.44 ± 4.38
	Handball	10.25 ± 5.03	10.20 ± 4.25#	9.00 ± 5.34#
	Swimming	7.40 ± 4.40	6.64 ± 3.56	5.00 ± 4.15

Data are presented as mean ± SD. TMD: Total Mood Disturbance. AU: Arbitrary units. # $p_{Bonferroni} \leq 0.05$ different to Athletics values. ^ $p_{Bonferroni} \leq 0.05$ different to Handball values.
